# The Role of Socio-Structural Factors in Influencing Feeding Intentions and Practices Among Mothers with Infants in Mthatha, South Africa

**DOI:** 10.3390/ijerph23010133

**Published:** 2026-01-21

**Authors:** Luviwe Lutotswana, Guillermo Alfredo Pulido-Estrada, Eric Maimela, Sibusiso Cyprian Nomatshila

**Affiliations:** 1 Faculty of Medicine and Health Sciences, School of Public Health, Walter Sisulu University, Mthatha 5117, South Africa; gpulidoestrada@wsu.ac.za (G.A.P.-E.); emaimela@wsu.ac.za (E.M.); 2 Society and Health Research Institute, Faculty of Medicine and Health Sciences, Walter Sisulu University, Mthatha 5117, South Africa

**Keywords:** breastfeeding, exclusive breastfeeding, socio-structural factors

## Abstract

Breastfeeding is universally regarded as the cornerstone of infant feeding, as it is the ideal infant feeding choice for optimal nutrition and development. Socio-structural factors of breastfeeding in child health play an important role in guiding women’s decisions on options to feed their babies. A cross-sectional study was conducted among mothers with infants aged 0–6 months in Mthatha, Eastern Cape, with the aim of assessing the role of socio-structural factors in shaping feeding intentions and practices among mothers with infants. Written Informed consent was obtained in accordance with the Declaration of Helsinki from the participants prior to data collection. Data was gathered with a validated designed questionnaire as well as analyzed using Social Sciences (SPSS) version 29. A total of 181 mothers were enrolled. Only 45.9% reported that they exclusively breastfed their babies, of which the highest proportion of exclusive breastfeeding (EBF) was observed among the 21–29 age group at 51.8%, and the lowest among those aged 20 years and below (3.6%). Marital status (*p* = 0.005) and employment status (*p* < 0.001) were significantly associated with exclusive breastfeeding, with higher EBF rates observed among married mothers and those who were self-employed. Both the EBF mothers and non-EBF mothers shared a common belief that colostrum was not beneficial for infants (*p* = 0.854), whereas their views differed significantly on the amount of water given to infants before they reached six months (*p* = 0.001). There was no significant relationship between EBF status and having a family member who had breastfed in the past six months (*p* = 0.815); also, a weak association was noted for having a friend who had breastfed recently (*p* = 0.057). The difference in EBF practice between those receiving antenatal care (ANC) breastfeeding education and those not receiving it was not statistically significant (*p* = 0.591). A statistically significant association was found between the support level and exclusive breastfeeding status (*p* < 0.001). This study highlights that the successful practice of exclusive breastfeeding (EBF) is strongly associated with high levels of social support. Interventions are needed to engage active partners, family members, and community members in creating a supportive environment for breastfeeding mothers.

## 1. Introduction

The World Health Organization (WHO) defines exclusive breastfeeding (EBF) as infants consuming only human milk, with the exception of oral rehydration solutions (ORS), drops, or syrups [1]. EBF is generally recommended as the optimal form of infant feeding during the first six months of life because of its health benefits for both the mother and the child. [2]. Exclusively breastfed infants have superior cognitive developmental scores, have a lower risk of gastrointestinal and respiratory disorders, and are less likely to acquire lifetime obesity and diabetes [3]. Furthermore, EBF for the full six months, as advised, reduces the risk of diarrheal illness by 50 percent, which is one of the primary causes of infant mortality [4].

Exclusive breastfeeding coverage still does not meet the target to increase EBF rates to at least 50% by 2025, which was set by the United Nations (UN) policy brief on The UN Decades of Action on Nutrition, due to various factors. Globally, about 40% of infants are exclusively breastfed, and this is expected to rise to 50% by 2025 [5]. Only 41% of infants worldwide are exclusively breastfed for the first six months, with South Africa’s EBF rate being only 32% [6].

Mothers ultimately decide whether to exclusively breastfeed their children. However, several factors affect EBF practice. Among these are maternal employment, parity, maternal perception of the quantity of human milk, maternal education, antenatal care (ANC) visits, postnatal care (PNC) utilization, maternal health, and birthplace are the most frequently associated factors [7].

There are now a variety of programs for supporting breastfeeding that may target pregnant women, their partners, family members, the health service, and/or broader communities. The WHO/United Nations International Children’s Emergency Fund (UNICEF) and the Baby-Friendly Hospital Initiative (BFHI), commonly known as the Baby-Friendly Initiative (BFI), have been shown to be the most effective healthcare interventions for encouraging breastfeeding initiation. The BFHI/BFI program is a complete, systematic approach that encompasses organizational change. It comprises the implementation of the Ten Steps to Effective Breastfeeding, which include staff training rules, breastfeeding promotion and support, restriction of the prescription and use of infant formula, prohibition of the use of teats and pacifiers, and keeping mothers and newborns together [8]. Mothers who opt for formula feeding are advised to strengthen the use of the spoon and cup for feeding and promotion of personal hygiene during preparation of formula, while all mothers on EBF or exclusive formula feeding (EFF) are advised not to give newborns any food or fluids other than human milk or infant formula in the first six months of life, unless medically indicated [9]. Exclusive breastfeeding promotion is also carried out through World Breastfeeding Week, commemorated every year from August 1 to 7 by more than 120 countries worldwide, to encourage breastfeeding and enhance the health of infants globally [10]. Despite global recommendations, there has been limited research on the association between breastfeeding-related knowledge and breastfeeding choice in KSD.

## 2. Materials and Methods

The current study was cross-sectional employing quantitative methods, conducted to assess the association between breastfeeding knowledge and breastfeeding choices among mothers in Mthatha, South Africa.

King Sabata Dalindyebo (KSD) Local Municipality is situated in the inland of Eastern Cape Province in South Africa. The municipality comprises Mthatha and Mqanduli, both of which cover an area of 3027 km^2^ and have a population of 476,558. The racial makeup is 98% Black Africans, 0.8% mixed-race individuals, and 0.3% white individuals [11]. The target population of this study consisted of mothers aged 16 to 35 years residing in KSD with children aged 0 to 6 months.

A two-stage cluster design was employed, in which, at the initial stage, three health facilities were randomly selected from the list of Health centers within the KSD municipality. A total of 61 participants were selected from each of the two facilities, with 59 participants chosen from one facility. Subsequently, during the second stage, a predetermined number of women were selected using simple random sampling from each clinic in proportion to the count of women with infants aged 0–6 months

The sample size (*n*) of the study population was calculated taking into consideration the following aspects:

- Expected proportion: *P* = 32% (Prevalence of exclusive breastfeeding practices in South Africa)
- Confidence Level: 95% (*Z_α_* = 1.96)
- *e*: The maximum error admitted by the researcher: *e* = 7% [12]

$$n=\frac{Z_{\alpha }^{2}\times P(100-P)}{e^{2}}$$

$$n=\frac{{\left(1.96\right)}^{2}\times 32(100-32)}{7^{2}}=\frac{3.84\times 2176}{49}=171$$

To reduce the nonresponse rate, 5% of this sample was added. Following this, the final sample size for the study consisted of 181 participants.

The sample size for the study was determined using Cochran’s formula to obtain a required sample size of 171 participants. The final sample size was 181, accounting for 5% non-response rate.

Written Informed consent was obtained in accordance with the Declaration of Helsinki from the participants prior to data collection. Written informed consent, providing full details about the study and its reporting channels, was obtained from the participants. The study included all consenting mothers aged 16 years and above with babies between 0 and 6 months of age. Mothers who were incapacitated were excluded from the study.

A validated structured questionnaire was used to gather data, which had been adopted from Minas (2016) [13] measuring key variables, such as demographics, breastfeeding socio-structural factors, and infant feeding practice. The questionnaire was tested in a small-scale population, not participating in the current study, and was adjusted accordingly. The questionnaire was also translated into the local language, IsiXhosa, and tested in a small-scale population that did not participate in the current study. The questionnaire was administered by the researcher, who completed the questionnaire based on the answers received from the participants.

All data were coded to safeguard the participants’ identities. After being cleaned and double-checked, the data were input into the Statistical Package for the Social Sciences (SPSS) version 29 on a password-protected personal computer located in a cloud repository and accessible only to the researcher. To measure breastfeeding knowledge, the level of agreement with key breastfeeding knowledge statements was assessed and compared between mothers who engaged in EBF and those who did not. Responses were measured on a 5-point Likert scale, where a score of 1 represented “totally disagree” and 5 “totally agree”, with the statement. To identify the association between the socio-demographic variables with exclusive breastfeeding, a chi-square test or Fisher’s exact test was used. A *p*-value of less than 0.05 was considered statistically significant, indicating a relationship between the identified variables. To compare the means between groups (EBF and NON-EBF), a *t*-test for independent samples was used if the variable followed a normal distribution, or a Mann–Whitney test if not. To assess the perceived social support for breastfeeding, participants responded to four Likert-scale items related to support from healthcare providers, family members, partners, and close friends. Each item was rated on a 5-point scale. Support for breastfeeding was assessed using four 5-point Likert-scale items that captured support from healthcare providers, family members, spouses/partners, and close friends or relatives. The total score (range 4–20) was calculated by summing responses across the four retained items, with higher scores reflecting greater perceived support. Based on the score distribution, support was classified as low (≤12), moderate (13–16), or high (≥17).

## 3. Results

### 3.1. Participant Characteristics

This study sample consisted of 181 women with infants aged 0–6 months residing in KSD Municipality. Most participants were between 21 and 29 years old (43.6%), single (71.3%), and had a secondary educational level (53%). Additionally, 51.4% of participants were unemployed (Table 1).

### 3.2. Association Between Sociodemographic Characteristics and Exclusive Breastfeeding (EBF)

The overall proportion of mothers who were exclusively breastfeeding was 45.9%. The highest proportion of EBF was observed among mothers aged 21–29 years (51.8%), followed by those aged 30 and above years (44.6%), as shown in Table 2. Marital status and employment status were significantly correlated with EBF (*p* = 0.005 and *p* = 0.000, respectively), with the highest rates of EBF found among married and self-employed mothers. There was a statistically significant association between maternal age group and EBF (*p* < 0.001). Religion and education level were not statistically significantly correlated with EBF.

### 3.3. Breastfeeding-Related Knowledge and Exclusive Breastfeeding

EBF mothers demonstrated better knowledge compared to non-EBF mothers in five of the seven statements, that is, regarding human milk being the best for new-born babies, initiation of breastfeeding within 1 h of delivery, regarding the recommended 2-years’ breastfeeding duration, importance of breastfeeding to infants with diarrhea and, not giving any other food except human milk to babies before the age of 6 months, including water or other liquids (Table 3).

No significance was observed for the statement that a mother should breastfeed 8 to 10 times in 24 h, that is, 8 to 10 times daily during the breastfeeding period.

Regarding the misconception “colostrum should be expressed and discarded”, both groups had similarly low agreement (means near 2.5), indicating a shared level of confusion or mixed beliefs (*p* = 0.854).

### 3.4. Association Between Previous Breastfeeding Experience and Exclusive Breastfeeding Status

A mother’s history of being breastfed was not a significant predictor of exclusive breastfeeding, as shown in Table 4. Similarly, there was no significant association between EBF status and having a family member who had breastfed in the past six months, with approximately 30.9% of participants reporting such exposure. The difference in EBF practice between those who received ANC breastfeeding education and those who did not was not statistically significant. A borderline association was noted for having a friend who had breastfed recently (*p* = 0.057), where 41.0% of EBF mothers reported such an experience compared to 27.6% among non-EBF mothers.

### 3.5. Association Between Support Level and Exclusive Breastfeeding

A statistically significant association was found between support level and exclusive breastfeeding status (Table 5). Among participants with high support, a greater proportion reported practicing exclusive breastfeeding (*p* < 0.001, Fisher’s exact test).

## 4. Discussions and Limitations

This study assessed the association between breastfeeding socio-structural factors and feeding choice in mothers and made the following key findings: the rate of EBF was 45.9%; the highest proportion of EBF was observed among the 21–29 age group, and the lowest among those aged 20 years and below; EBF was most common in married mothers compared to single mothers; Mothers who were self-employed had the highest proportion practicing EBF, followed by employed mothers; and EBF mothers demonstrated better knowledge of recommended breastfeeding practices compared to non-EBF mothers.

The EBF rate observed in the current study is slightly greater compared to the global rate, which currently stands at only 44% of infants being breastfed shortly after birth, and 40% of those under six months old are exclusively breastfed [14]. In a study undertaken in South Africa, it was found that the EBF rate was just 32% [6]. The rate of EBF in this current study was determined by exclusive breastfeeding status at the time of the study, including participants whose babies had not reached the age of 6 months. Therefore, there is a possibility that the rate of EBF measured at 6 months—actual EBF rate—could be lower than the one measured before 6 months, as was carried out in the current study.

The lowest proportion of EBF was observed among those aged 20 years and below, which may be attributed to the fact that the majority in this age group were still in secondary school, and some younger mothers lacked adequate knowledge related to breastfeeding. According to a study by [15], adolescents who receive antenatal education are more likely to maintain, intend, and feel confident about breastfeeding and in comparison to women aged 20–29 (36.4%) and those over 30 (45.0%), adolescent mothers are the least likely to exclusively breastfeed their infants in the first 6 months, with percentages falling below 25% [15]. In another study, it was stated that younger mothers are less likely than older mothers to exclusively breastfeed or to exclusively breastfeed for a long duration [16].

A stratified review of the data revealed that the positive association between marital status and exclusive breastfeeding persisted across age groups. Additionally, self-employed mothers consistently demonstrated higher exclusive breastfeeding rates than unemployed mothers within both secondary and post-secondary education categories. Similarly, mothers with adequate breastfeeding knowledge exhibited higher EBF prevalence across all age groups. Although the study was not powered for formal interaction testing across multiple subgroups, these patterns suggest that social support, flexible work structures, and breastfeeding knowledge jointly reinforce breastfeeding behaviors.

When examining support levels by marital status and EBF status, married mothers consistently reported higher partner and household support, and this support was more evident among those who exclusively breastfed. These descriptive patterns suggest that the protective effect of marital status on breastfeeding may be partially mediated by greater access to consistent emotional and practical support within the household. Although the sample size limited formal interaction testing, the observed trends reinforce the importance of social and partner support in sustaining exclusive breastfeeding practices.

Another study [17] concur that there is a relationship between family support and the provision of EBF. This finding is consistent with existing evidence suggesting that support from family members, particularly husbands, plays a critical role in influencing whether or not exclusive breastfeeding is achieved [18].

A significant association was observed between employment status and EBF, where mothers who were self-employed had the highest proportion practicing EBF, followed by employed mothers. This could be due to workplaces not being breastfeeding-friendly and not having lactating spaces, whereas self-employed mothers had the flexibility of working in their own spaces. Another study also highlighted that continuing breastfeeding after returning to work poses significant challenges for employed mothers, with most requiring adequate support to sustain the practice [19]. Employers play a critical role in establishing supportive systems and providing suitable workplace facilities that enable mothers to express and store their milk [19]. Another study agreed that some mothers were able to successfully manage the conflicting demands of work and childcare due to the flexibility of informal employment [20].

Breastfeeding knowledge in this sample appeared to be shaped more by maternal characteristics and informal exposure than by antenatal education. Given that participants did not receive specific education on the value of colostrum and that colostrum content is often inconsistently addressed in antenatal programs, this gap may partly explain the limited impact of antenatal education observed in our findings. Higher knowledge scores were observed among older mothers, those with higher education levels, and those who had family or friends who breastfed, indicating that social learning and prior exposure may reinforce breastfeeding knowledge and confidence. Mothers who exclusively breastfed exhibited better breastfeeding-related knowledge of recommended breastfeeding practices compared to non-EBF mothers. This suggests a knowledge gap about exclusive breastfeeding. Mothers who are well-versed in breastfeeding and who comprehend that human milk is given without supplementary food or beverages are more likely to breastfeed because their behavior will be influenced by their knowledge when making decisions to exclusively breastfeed [18].

In this study, there was no association between previous breastfeeding experience and exclusive breastfeeding status; that is, there was no significant association between EBF status and having a family member who had breastfed in the past six months, with approximately 30.9% of participants reporting such exposure. Although not statistically significant at the *p* < 0.05 level, the data suggest that being breastfed as a child and having a friend who breastfed in the past 6 months may also be associated with a greater likelihood of EBF at the time of the interview, which might have been captured with a larger sample size.

Out of the total sample, most participants reported receiving health education on breastfeeding during antenatal care (ANC) visits, including 79.5% of mothers who were exclusively breastfeeding (EBF) and 82.7% of those who were not. The difference in EBF practice between those who received ANC breastfeeding education and those who did not was not statistically significant. This suggests that while most participants were exposed to breastfeeding information during ANC, this exposure alone was not associated with a higher likelihood of practicing exclusive breastfeeding in this sample, indicating that routine ANC counseling may be insufficient in terms of content, intensity, or delivery method. Strengthening ANC breastfeeding counseling through more structured, practical, and continuous support, alongside community-based peer support programs, may improve knowledge transfer and breastfeeding outcomes. In contrast to other studies, where a higher percentage of EBF practice was observed among mothers who had ANC visits than those who had no ANC visits [21,22,23].

A statistically significant association was found between support level and exclusive breastfeeding status. The composite support score integrated several sources of social and healthcare-related assistance, including support from partners, family, and healthcare providers. Patterns observed across the retained domains suggest that social and relational networks continue to play a crucial role in promoting exclusive breastfeeding, particularly among married and family-supported mothers. Among participants with high support, a greater proportion reported practicing exclusive breastfeeding, compared to those with moderate support and none with low support. These findings suggest that a greater level of support is positively correlated with the likelihood of practicing exclusive breastfeeding. A study conducted in South Africa also found that for first-time moms, social and practical support is essential for starting and sustaining exclusive breastfeeding during the first six months of life [22]. Another study [24] concurs that the level of support from partners, friends, and family is a predictive factor for EBF.

An important finding of this study is the relatively high rate of exclusive breastfeeding among single mothers. Although single mothers reported lower partner support, many indicated strong support from family members and peers, suggesting that alternative support networks may compensate for the absence of a spouse/partner. This aligns with evidence that extended family and community support can be equally influential in sustaining breastfeeding practices, particularly in settings where grandmothers and female relatives play key caregiving roles.

This study’s limitations include the fact that the cross-sectional design limits the ability to draw causal inferences between the identified socio-structural factors and exclusive breastfeeding practices. The observed relationships represent associations at a single point in time and may not accurately reflect temporal or causal effects. Secondly, the study relied on self-reported data for breastfeeding practices and related variables, which may be subject to recall bias and social desirability bias. Some participants may have overreported exclusive breastfeeding practices due to cultural expectations or perceived social norms. Another limitation includes the measurement of exclusive breastfeeding status at the time of the study, which included participants whose infants had not yet reached six months of age. Consequently, the reported rate of exclusive breastfeeding may overestimate the actual prevalence maintained through six months, as breastfeeding exclusivity often declines with infant age.

## 5. Conclusions

The current study demonstrates that exclusive breastfeeding (EBF) practices are significantly influenced by a combination of demographic, social, and knowledge-based factors. Notably, the study emphasizes the significance of social and family support, particularly for married and self-employed mothers, as well as the impact of prior exposure to breastfeeding through family and friends on mothers’ knowledge and confidence in breastfeeding. However, factors such as previous personal or familial breastfeeding experience and the receipt of antenatal health education did not demonstrate a significant statistical relationship with EBF outcomes in this sample, despite their noted importance in other studies. This study emphasizes that while knowledge gained from health education is foundational, it is insufficient on its own; practical and social support from partners, family, and friends emerges as the critical, actionable factor that positively influences a mother’s decision and ability to sustain exclusive breastfeeding.

Despite the positive outcomes observed in this study, several limitations—such as the cross-sectional design and self-reported data—suggest that further research is needed to explore the causal pathways linking socio-structural factors to breastfeeding practices.

Given the significant role of social support in sustaining breastfeeding practices, interventions aimed at enhancing partner involvement and increasing family and community support networks could be beneficial. Additionally, workplace policies that accommodate breastfeeding mothers, such as providing lactation spaces and flexible work hours, could further support breastfeeding success, particularly for employed mothers. Strengthening antenatal care counseling, with an emphasis on practical, structured, and continuous support, may also improve breastfeeding outcomes by ensuring that all mothers receive the necessary information and confidence to exclusively breastfeed.

Ultimately, the findings from this study reinforce the need for a multifaceted approach to promoting exclusive breastfeeding, one that combines health education, social support, and supportive workplace and policy environments to ensure that more mothers can practice exclusive breastfeeding for the first six months of life.

## Figures and Tables

**Table 1:** Sociodemographic attributes of a sample of women with infants from 0 to 6 months of age in KSD Municipality.

Sociodemographic	Frequency	Percent (%)
Age group		
- 20 and below	34	18.8
- 21–29	79	43.6
- 30 and above	68	37.6
Total	181	100.0
Marital status		
- Single	129	71.2
- Married	47	26.0
- Separated/Widowed	5	2.8
Total	181	100.0
Religion		
- Orthodox Christian	144	79.6
- Catholic Christian	12	6.6
- Protestant Christian	11	6.1
- Other	14	7.7
Total	181	100.0
Highest level of education		
- Primary level	1	0.5
- Secondary level	95	52.5
- Post-secondary level	85	47.0
Total	181	100.0
Employment status		
- Not employed	93	51.4
- Self-employed	40	22.1
- Employed	48	26.5
Total	181	100.0

**Table 2:** Association between sociodemographic attributes and exclusive breastfeeding (EBF) choice.

Sociodemographic Variables	Not EBF n (%)	EBF n (%)	Total n (%)	*p*-Value
Age group				
- 20 and below	31 (31.6)	3 (3.6)	34 (18.8)	0.000
- 21–29	36 (36.7)	43 (51.8)	79 (43.6)	
- 30 and above	31 (31.6)	37 (44.6)	68 (37.6)	
Marital Status				
- Single	79 (80.6)	50 (60.2)	129 (71.3)	0.005
- Married	18 (18.4)	29 (34.9)	47 (26.0)	
- Separated/Widowed	1 (1.0)	4 (4.8)	5 (2.8)	
Religion				
- Orthodox Christian	80 (81.6)	64 (77.1)	144 (79.6)	0.327
- Catholic Christian	7 (7.1)	5 (6.0)	12 (6.6)	
- Protestant Christian	3 (3.1)	8 (9.6)	11 (6.1)	
- Other	8 (8.2)	6 (7.2)	14 (7.7)	
Education Level				
- Primary	1 (1.0)	0 (0.0)	1 (0.6)	0.498
- Secondary	54 (55.1)	41 (49.4)	95 (52.5)	
- Post-secondary	43 (43.9)	42 (50.6)	85 (47.0)	
Employment Status				
- Not employed	61 (62.2)	32 (38.6)	93 (51.4)	0.000
- Self-employed	10 (10.2)	30 (36.1)	40 (22.1)	
- Employed	27 (27.6)	21 (25.3)	48 (26.5)	

**Table 3:** Comparison of knowledge-related breastfeeding statements between EBF and Non-EBF mothers.

Statement	EBF Mean (SD)	Non-EBF Mean (SD)	Mean Difference	*p*-Value
Human milk is the best for a newborn baby.	4.51 (0.53)	4.22 (0.49)	0.28	<0.001
A mother should start breastfeeding within one hour of delivery.	3.80 (0.89)	3.48 (0.71)	0.32	0.009
Colostrum should be expressed and discarded.	2.52 (1.32)	2.55 (1.09)	−0.03	0.854
A mother should breastfeed her child about 8 to 10 times in 24 h.	2.89 (0.64)	2.97 (0.60)	−0.08	0.402
A mother should breastfeed her child for at least two years.	2.76 (1.16)	1.93 (0.75)	0.83	<0.001
An infant with diarrhea should be breastfed.	4.31 (0.90)	3.77 (1.10)	0.55	<0.001
Water or other liquids can be given before 6 months.	3.46 (0.89)	3.94 (0.53)	−0.48	<0.001

**Table 4:** Association between previous breastfeeding experience and breastfeeding status of participants.

Previous Breastfeeding Experience	EBF Yes n (%)	EBF No n (%)	Total n (%)	*p*-Value
Breastfed as a child				
Yes	61 (73.5)	84 (85.7)	145 (80.1)	0.069
No	7 (8.4)	7 (7.1)	14 (7.7)	
Don’t know	15 (18.1)	7 (7.1)	22 (12.2)	
Family breastfeeding in the past 6 months				
Yes	24 (28.9)	32 (32.7)	56 (30.9)	0.815
No	58 (69.9)	65 (66.3)	123 (68.0)	
Don’t know	1 (1.2)	1 (1.0)	2 (1.1)	
Friend breastfeeding in the past 6 months				
Yes	34 (41.0)	27 (27.6)	61 (33.7)	0.057
No	49 (59.0)	71 (72.4)	120 (66.3)	
Health education on breastfeeding during ANC				
Yes	66 (79.5)	81 (82.7)	147 (81.2)	0.591
No	17 (20.5)	17 (17.3)	34 (18.8)	

**Table 5:** Association between the availability of support and Exclusive Breastfeeding.

Support Level	EBF Yes n (%)	EBF No n (%)	Total n (%)
Low	0 (0.0)	1 (1.0)	1 (0.6)
Moderate	9 (10.8)	41 (41.8)	50 (27.6)
High	74 (89.2)	56 (57.1)	130 (71.8)
Total	83 (100.0)	98 (100.0)	181 (100)

## Data Availability

The collected data were used for the purpose of this study and are stored in a password-protected cloud repository, accessible only to the researcher and supervisor. All collected data is available on request.

## Abbreviations

The following abbreviations are used in this manuscript:

EBF	Exclusive Breastfeeding
KSD	King Sabatha Dalindyebo
ORS	Oral rehydration solutions
WHO	World Health Organization
UN	United Nation
ANC	Antenatal care
PNC	Postnatal care
UNICEF	United Nations International Children’s Emergency Fund
BFHI	Baby-Friendly Hospital Initiative
